# Supplementary material for: Predicting visual function by interpreting a neuronal wiring diagram
Source: Nature. 2024 Oct 2;634(8032):113–23. doi: 10.1038/s41586-024-07953-5 (PMC11446822; doi:10.1038/s41586-024-07953-5)
Supplement: Supplementary file 6 — CRF and ERF predictions for individual TmY4 and TmY9 cells. Analogous to Supplementary Data 3, but for TmY target types. Shown are the top four monosynaptic pathways, the strongest pathway passing through each of the top ten intermediary types (ranking from Extended Data Fig. 7), and the trisynaptic pathway Tm1–TmY–Dm3–TmY (see the section entitled Prediction of spatial normalization). [file 41586_2024_7953_MOESM6_ESM.zip › DataS4/TmY9q/720575940616902086.pdf]

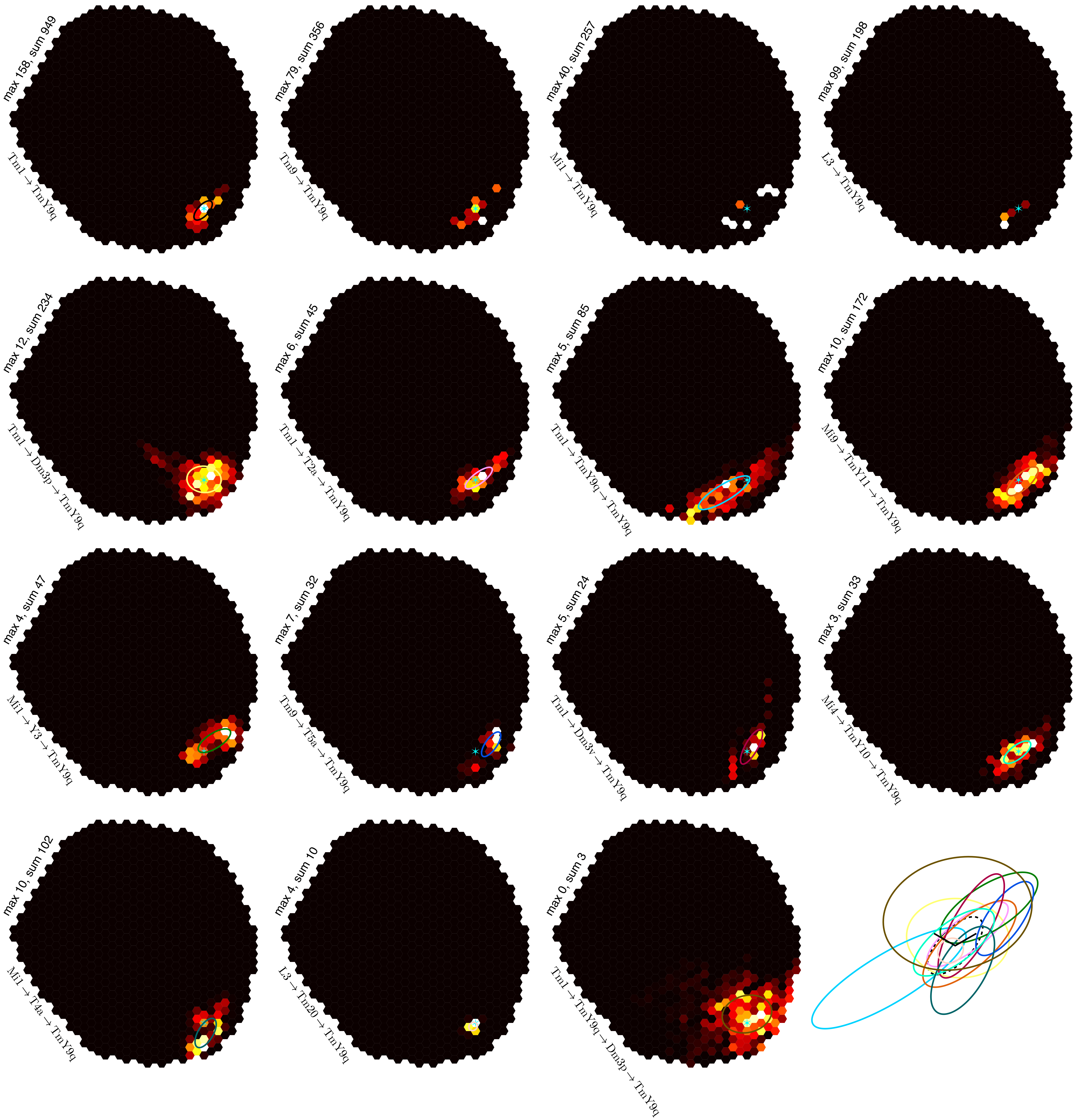
max 158, sum 949
Tm1 → TmY9q
max 79, sum 356
Tm9 → TmY9q
max 40, sum 257
Mi1 → TmY9q
max 99, sum 198
L3 → TmY9q
max 12, sum 234
Tm1 → Dm3p → TmY9q
max 6, sum 45
Tm1 → T2a → TmY9q
max 5, sum 85
Tm1 → TmY9q → TmY9q
max 10, sum 172
Mi9 → TmY11 → TmY9q
max 4, sum 47
Mi1 → Y3 → TmY9q
max 7, sum 32
Tm9 → T5a → TmY9q
max 5, sum 24
Tm1 → Dm3v → TmY9q
max 3, sum 33
Mi4 → TmY10 → TmY9q
max 10, sum 102
Mi1 → T4a → TmY9q
max 4, sum 10
L3 → Tm20 → TmY9q
max 0, sum 3
Tm1 → TmY9q → Dm3p → TmY9q
